# The determinants of sovereign risk premiums in the UK and the European government bond market: the impact of Brexit

**DOI:** 10.1007/s10368-022-00535-8

**Published:** 2022-05-31

**Authors:** Samir Kadiric

**Affiliations:** Research Associate at European Institute for International Economic Relations (EIIW), University of Wuppertal, Rainer-Gruenter-Str. 21, 42119 Wuppertal, Germany

**Keywords:** Asset pricing, Government bond yield spreads, Risk premium, UK, Europe, Brexit, E43, E44, F36, G12, G15

## Abstract

This paper analyzes recent developments in the British and European government bond markets with reference to the UK’s decision to leave the European Union. The two main goals of the study are, firstly, to examine whether the Brexit referendum result has affected the risk premium and, secondly, whether there are any changes in risk pricing following the referendum. The paper finds a significant impact of the Brexit referendum on the risk premium in selected economies. Furthermore, the results suggest that there is a considerable change in risk pricing after the announcement of the referendum result. Credit default risk and the risk aversion play a much important role in the post-referendum period than they did prior to the vote, particularly in the UK.

## Introduction

On June 23, 2016, the British people voted in a referendum for the United Kingdom (UK) to leave the European Union (EU)—an event which became widely known as “Brexit.” This decision in favor of leave was, broadly speaking, unexpected by most observers. Hence, Brexit represents a unique shock that limits the extent to which previous analyses can be used to understand its effects. During 47 years of membership, a set of complex relationships between the UK and the economies of other EU member states developed. Thus, the departure of the UK from the EU would cause a significant loss for both sides.

Against this background, the aim of this study is to analyze recent developments in the UK and the European government bond markets with reference to the UK’s decision to leave the EU and to discuss their implications for both policymakers and economists. Sovereign bond yields play a key role in the transmission process of the central bank’s monetary policy. Moreover, they are generally used as a benchmark to price key interest rates in financial markets, for asset allocation and asset pricing purposes (see ECB (2014a)). Furthermore, from a long-term policy perspective, it is important to understand the main drivers of sovereign risk in order to find an appropriate and effective policy mix and so meet the challenges of Brexit and its aftermath in coming years.

This paper is related to the literature which focuses on the effects of the Brexit referendum result on financial markets. Using composite indicators of financial integration, Hoffmann et al. (2020) find that the announcement of the Brexit referendum result led to a decline of the level of financial integration in Europe, although not as strong as during the financial crisis. The estimation results in Belke et al. (2018) reveal significant evidence that an increase in the likelihood of Brexit had a strong negative effect on stock prices, with the largest effect found for UK stocks. In addition, Hill et al. (2019) show that financial and consumer-facing sectors had the highest exposure to the uncertainty surrounding the Brexit vote. Schiereck et al. (2016) analyze stock and credit default swap (CDS) market reactions in the UK and the EU around the time of the Brexit vote (CDS indicate the price of default risk in financial markets). They find that a short-run drop in stock prices to the referendum announcement was more pronounced than to the bankruptcy of the Lehman Brothers, particularly for EU banks, although an increase in CDS spreads is relatively small, compared to Lehman bankruptcy.

Using an event study methodology, Ramiah et al. (2017) confirm the finding that most economic sectors reacted negatively to the referendum result as indicated by negative abnormal returns; however, the banking sector was affected the most. Using a two-stage estimation process, a subsequent study by Davies and Studnicka (2018) finds that firms with global value chains more strongly oriented toward Europe perform worse than the market as a whole while larger firms seemed to ride out the turmoil of Brexit much more easily than the average firm. Moreover, they find that the market’s reaction to the announcement of the referendum result was persistent. Moreover, Breinlich et al. (2018) analyze the short-run effects of Brexit by studying stock market reactions. Their results suggest that exchange rate movements and investors’ expectations of an economic slowdown were the main driver of stock market reactions to the referendum result.

Analyzing the effects of the Brexit referendum on the exchange rate, Belke et al. (2018) assessed that an increase in the probability of Brexit decreases the value of the British pound. Caporale et al. (2018) find that the Brexit referendum led to a significant change in the degree of persistence of the FTSE 100 Implied Volatility Index and of the British pound’s implied volatilities vis-à-vis the euro and the US dollar, respectively. Studying the effect of the Brexit vote on intraday currencies, Dao et al. (2019) observe a substantial decrease in volatility transmission between British sterling and the euro following the Brexit vote due to lower levels of market integration. Pilbeam (2019) shows that the Brexit referendum caused a significant depreciation of the British pound against both the US dollar and the euro. Moreover, analyzing the impact of Brexit-related news on the spot exchange rate of the British pound, Korus and Celebi (2019) find that “bad” Brexit news (higher probability of hard Brexit) are associated with a depreciation whereas “good” Brexit news appreciates the pound sterling against the euro.

Focusing on corporate bond markets in the UK as well as in the euro area (EA), Kadiric and Korus (2019) find that the Brexit referendum result had a significant impact on credit spreads. The announcement of the referendum result led to increasing yield spreads in both markets. Furthermore, differentiating between financial and non-financial economic sectors, their results indicate that the impact of Brexit is stronger for financials than for non-financials, especially in the EA where corporate bond spreads in the non-financial sector were hardly or not at all affected by the referendum result. Following up on this work, Welfens et al. (2019) further differentiate corporate bond market in the UK by introducing AA and BBB rating categories as representatives of a higher and lower credit rating quality. Their results suggest that market participants did not make a distinction between AA- and BBB-rated bonds, since corporate bond spreads were affected by the announcement of the referendum result irrespective of the rating category.

Belke et al. (2018) elaborate on the impact of Brexit on financial markets, including 10-year government bond yields and sovereign CDS for 10-year bonds. Confirming the results presented by the Bank of England (2016), they find that an increase in the Brexit probability led to a strong decrease in long-term interest rates for the UK and additional “risk-free” countries, respectively, although their results indicate that sovereign CDS for 10-year bonds increased in the UK due to Brexit. Chadha et al. (2018) confirm these results. Focusing on the long-term gilt yield, they find that bond yields decline in the direct aftermath of the referendum. Their findings suggest that Brexit-related uncertainty put upward pressure on UK government bond yields. However, the anticipation of expansionary monetary policy measures appears to have offset any change in risk premiums. Thus, using long-term yields might not be an appropriate way to capture and analyze risk conditions in the government bond markets (see Bernoth et al. (2012) and Gale and Orszag (2003)).

On account of this, the present study uses yield spreads as an indicator of a risk premium, calculated as the difference between the respective government bond yield and a “risk-free” rate; in this case, the OIS rate is used. The risk premium is expected to embody the risk conditions exposure of the UK and selected EA countries. The frequency of data is daily, covering the period from October 1, 2014, to March 29, 2019. The choice of the risk premium determinants is mainly based on the theoretical background and on the existing literature in this field. This analysis addresses several questions: firstly, did the UK’s decision to leave the EU (Brexit) have an immediate direct effect on sovereign risk in the UK and other EA countries? Secondly, has Brexit triggered some changes in the pricing of sovereign risk due to expected challenges in the future economic development in the UK and selected EA countries?

This paper offers several contributions to the existing literature on the determinants of sovereign bond yield spreads. Firstly, this is to my knowledge the first study that focuses on the potential effects of the Brexit referendum result on risk premiums in the UK and European government bond markets. Secondly, it extends the existing literature on the effects of Brexit on financial markets. Thirdly, analyzing risk premiums in the UK and the EA government bond markets simultaneously allows for direct comparison of Brexit effects in those markets. Fourth, estimating the period before and the period after the announcement of the Brexit referendum result enables an analysis of potential changes in the investors’ risk assessment. Finally, this paper employs a newly developed regional risk aversion variable in order to capture the willingness of investors to bear county-specific risks.

There are three key findings of this paper. Firstly, the announcement of the Brexit referendum result led to an immediate increase of the risk premium in the UK and some other selected European government bond markets. Secondly, the results suggest that there is a considerable change in the importance of the determinants of sovereign bond spreads due to the change in the risk pricing triggered by the Brexit referendum result. This holds particularly for the UK, where the credit default risk and risk aversion play a much more important role in the post-referendum period (the period after the referendum and before the day of Brexit implementation on December 31, 2020) than they did before. Thirdly, the empirical results indicate that using regional rather than international risk aversion might be more appropriate in order to capture investors’ risk assessment, especially when analyzing euro area countries.

The reminder of the paper is organized as follows. Section 2 offers the theoretical background and gives an overview of the related literature. Section 3 presents the data used in this study. Section 4 is the core of the paper and analyzes the impact of Brexit, pricing developments, and their implications. Section 5 provides an additional robustness analysis, while Sect. 6 concludes.

## Theoretical background and related literature

There is an extensive, financially rewarding and outstanding body of empirical literature that deals with the determinants of sovereign bond yield spreads in the euro area. The establishment of the European Monetary Union (EMU) has eliminated the exchange rate risk as a source of market segmentation and a key obstacle to financial integration between participating member states. Some other aspects and sources of risk, such as expected inflation and central bank credibility (see, Haugh et al. (2009)), were also either eliminated or minimized. Without an exchange rate risk, but still including different sovereign issuers, the euro area provides an excellent experimental field for studying country risk and its determinants. The yield differentials of euro area government bonds against the generally used German benchmark have declined radically after the start of the monetary union. Codogno et al. (2003), Pagano and von Thadden (2004), Geyer et al. (2004), and Gomez-Puig (2006) find an overwhelming convergence of sovereign bond yields as a strong indication of market integration. Although small, the nevertheless non-negligible variable yield differentials for sovereign debt, which vary both across countries and over time, indicate that euro area bonds are still not perfect substitutes.^1^ Reorganization of the market structure has changed portfolio composition and the trading strategy of investors, affecting both pricing and trading activity in the euro area bond markets (see Blanco (2001) and Gómez-Puig (2008)).

The predominant commonality of the previous studies lies in their use of three main explanatory determinants which should reflect investors demand for higher return—in a relation to that of a benchmark—as a compensation for the bearing of a higher risk. Two of them, namely the credit and the liquidity risk, are country-specific risks, whereas risk aversion represents an investors’ related risk (see Codogno et al. (2003), Barrios et al. (2009), and ECB (2014a)). Using a model of portfolio choice, Bernoth et al. (2012) provide both a theoretical justification for, and empirical evidence of, the role of explanatory variables.

Whereas credit and liquidity risk are attributes of country-specific characteristics, international *risk aversion* reflects a global factor. It represents the willingness of investors to bear those county-specific risks (credit and liquidity). Therefore, risk aversion is coupled with expectations about the future state of an economy. In periods of increased market turmoil and high uncertainty, the risk aversion is higher; consequently, investors are less willing to bear an additional risk—since their primary source of income is already at risk—and are rebalancing their portfolio toward less risky and more liquid assets (see Manganelli and Wolswijk (2009), Sgherri and Zoli (2009)). The perception of risk (risk pricing) is adjusting to new economic conditions, inducing a higher sensitivity of the yield spreads on changes in credit and liquidity risk. Even if the “amount of risk” stays constant over time, the yield spreads could rise due to the shift in “price of risk” (see Barrios et al. (2009)). Thus, risk aversion can influence yield differentials per se but also via interaction with other variables.

There is a unanimous consensus in the literature that international risk aversion plays a crucial role in explaining the yield spreads in sovereign bond markets. The common finding in the literature is that euro area sovereign yield spreads strongly comove, particularly in the period before the financial and sovereign debt crises in Europe. This phenomenon is well observed and econometrically supported. Principal component analysis shows that the first principal component generally can explain more than 90% of the variation in sovereign bond yield series (see, e.g., Barrios et al. (2009), Manganelli and Wolswijk (2009), Favero et al. (2010), Gerlach et al. (2010)). These results confirm previous finding (e.g., Dungey et al. (2000), Codogno et al. (2003), and Geyer et al. (2004)) that a single time-varying common factor is a major driving force of variation in yield spreads. This common factor is strongly linked to international risk aversion. Since investors’ willingness to bear risk is not directly observable, international risk aversion is usually proxied by the yield spread between US corporate and government bonds or the implied volatility of S&P 500 index (VIX).

Manganelli and Wolswijk (2009) use the level of the short-term interest rate and argue that a low interest rate increases the incentives of investors to take on risk and therefore decreases yield spreads. Barrios et al. (2009) and Sgherri and Zoli (2009) focus on euro area sovereign risk during the last financial crisis. They confirm the former results that risk aversion still plays a major role in explaining the yield spreads in the euro area sovereign bond markets, although market concerns about debt sustainability rose with a deterioration in fiscal position. In addition, Haugh et al. (2009) emphasize the role of an interaction factor between risk aversion and fiscal position. They conclude that during the financial crisis, high international risk aversion magnified the effects of fiscal performance. One important consequence of the findings of Gerlach et al. (2010) is that international risk aversion can have large and rapid effects on government bond yield spreads. This effect is stronger and more striking in countries where underlying fundamentals are comparatively weak. In a more recent study, Gómez-Puig et al. (2014) use an exhaustive compilation of the variables in a sample of both central and peripheral European countries from January 1999 to December 2012 to estimate the government bond spreads. Their results confirm the significance of the global market sentiment and investors’ risk aversion in both periods, i.e., before and after the financial crisis. However, the marginal effects are far greater in the crisis period, particularly in the EMU peripheral countries, due to a “flight-to-quality” phenomenon in times of increased uncertainty. However, with the onset of the financial crisis and, later, of the sovereign debt crisis in Europe, other determinants of yield spreads have also gained in importance (see Barrios et al. (2009), Afonso et al. (2015a)).

*Credit risk* or default risk reflects the probability that the issuing country would not be able to service its obligations, at least partially (see Bernoth et al. (2012), and Manganelli and Wolswijk (2009)). In the event of imminent insolvency, the government could increase the taxes on interest income withheld at source or even negotiate a “haircut” on debt owed to the private sector (as in the case of the Greek government-debt crisis). One way or the other, there is a reduction in the investor’s return, so that the investor receives only a portion of his gross interest return or of the repayment of principal. Hence, since the security is subject to partial default risk, the investors demand a credit premium as a recompense for bearing the risk that a government could default and the investor not receiving his full interest payments or investment.

Thus, market participants can put pressure on governments by pricing different risks of default. Investors demand higher credit premiums for bonds of governments that follow unsound fiscal policy, forcing market discipline on them (see Manganelli and Wolswijk (2009)). Financial markets can penalize governments for a lack of fiscal discipline. Such a market force is particularly important in a monetary union, like the EMU, where the governments of the member states on the one hand can issue debt in their own right but on the other hand do not have any control on monetary policy (Schuknecht et al. (2009)). Therefore, the determinants of credit risk are typically related to the fiscal position and economic stance of the respective country.

In the previous studies, a wide range of fiscal and macroeconomic variables are used to proxy credit risk.^2^ Debt and deficit-to-GDP ratios are variables typically applied to describe the fiscal position of a country. To account for the forward-looking behavior of financial markets, several studies use expected rather than current or past fiscal fundamentals as determining variables (see, e.g., Heppke-Falk and Hüfner (2004), Haugh et al. (2009), Sgherri and Zoli (2009), Attinasi et al. (2010), Borgy et al. (2011), Bernoth et al. (2012), and D’Agostino and Ehrmann (2014)). Their results confirm the finding that using forward-looking data is important and can crucially affect the results. Amongst others, Gomez-Puig (2006), Manganelli and Wolswijk (2009), Arezki et al. (2011), Afonso et al. (2012), De Santis (2012), Afonso et al. (2015a), and Gärtner and Griesbach (2016) find that the sovereign credit rating plays an important role in explaining the changes in yield spreads, especially after the onset of financial crisis. The sovereign credit rating is linked to the long-term sustainability of a country’s finances and therefore influences the credit risk premium. An alternative way to account for the credit premium in government bond yield spreads is to use credit default swap (CDS). A sovereign CDS protects its holder(s) against financial losses in the case of a “credit event” of the CDS’ issuers. Thus, CDS represents a direct measure of the default risk. Barrios et al. (2009), Beber et al. (2009), Favero and Missale (2012), and Klose and Weigert (2014) find a statistically significant and economically sizable effect of sovereign CDS on yield spreads in the European government bond markets.

Focusing on the first years of the EMU, Codogno et al. (2003) find that fiscal variables play a role only when interacting with international risk aversion, while Geyer et al. (2004) find no effects of macroeconomic fundamentals. Analyzing the recent financial crisis, Barrios et al. (2009) and Mody (2009) conclude that before the crisis, country-specific factors were not important determinants of yield spreads. A subsequent study by De Grauwe and Ji (2013) supports these findings. An extensive convergence of sovereign bond yields after the start of the EMU and a weak evidence of the role of fiscal and macroeconomic fundamentals in explaining changes in yield spreads raised the question of the efficiency of the market discipline. In turn, Haugh et al. (2009), Manganelli and Wolswijk (2009), and Bernoth et al. (2012) affirm the role of fiscal variables both before and after the financial crisis. However, they all underline the finding that credit risk became a more important determinant in explaining yield spreads since the onset of the crisis. Amongst others, Attinasi et al. (2010), Gerlach et al. (2010), and Acharya et al. (2014) point to the crucial role of the banking sector in this process. The size of the banking sector, the announcement of bank rescue packages, and bank bailouts reinforce the risk transfer from the private to the public sector, leading to a revaluation of the credit risk by investors (bank-sovereign nexus).

Haugh et al. (2009), Barrios et al. (2009), von Hagen et al. (2011), and Borgy et al. (2011) stress the important role of risk aversion, arguing that in the crisis period, the yield spreads were to a large extent driven by changes in risk pricing. Their empirical results show that the markets penalized fiscal imbalances much more strongly since the onset of the financial crisis and, to an even greater magnitude, since the sovereign debt crisis. More recently, Delatte et al. (2017) assess the government bond spreads of five peripheral European countries (GIIPS) by applying a panel smooth threshold regression model. They confirm previous findings indicating regime-switch dynamics in these markets during the crisis. Sharp changes in the importance and pricing of the fiscal fundaments raised a question on the mispricing of credit risk. Arghyrou and Kontonikas (2012), Beirne and Fratzscher (2013), De Grauwe and Ji (2013), Aizenman et al. (2013), Gärtner and Griesbach (2016), and De Grauwe et al. (2017) likewise confirm these findings, supporting the relevance of contagion and multiple equilibria in this relationship. D’Agostino and Ehrmann (2014), using high-frequency proxies for market expectations about macroeconomic fundamentals and allowing for time-varying parameters, support the finding that changes in risk appetite have led to an under-pricing of credit risk prior to the global financial crisis and to either an over-pricing of risk or the presence of redenomination risk during the European sovereign debt crisis. The aspects of the redenomination risk in the European bond markets are further analyzed in Klose and Weigert (2014), De Santis (2015), and Klose (2019).

Pozzi and Wolswijk (2012), Aßmann and Boysen-Hogrefe (2012), Bernoth and Erdogan (2012), and Afonso et al. (2015b) use a time-varying approach, albeit different in modelling and specifications, that allows them to study the variation in the relationship between the determinant and bond yield spreads over time. In general, their results show the growing importance of credit risk in explaining yield differentials in the European sovereign bond markets, regardless of the role of risk aversion. The empirical results by Adam and Do Luca (2017) support the view that the pricing mechanism of the bond yield is not stable across periods. Conducting an analysis in both country-by-country and panel setups, Afonso and Jalles (2019) further scrutinize and corroborate the view of structural instability in the relationship between government bond yield spreads and their determinants.

From a general economic sense of risk and from a theoretical point of view, it is to be expected that for a less liquid asset, investors would demand a higher return as a financial compensation for bearing the risk of having to sell the asset at a lower price in relation to a respective benchmark. Nevertheless, the multifarious aspects make the concept of market liquidity hard to grasp. Following the definition provided by Gravelle (1999) and more generally by the Bank for International Settlements (1999), a market can be seen as liquid if a desirable volume of transactions can be traded immediately and quickly without any or at the best only with a small impact on current as well as on the subsequent price of the asset.

The prior studies have shown that capturing and measuring the effects of *liquidity risk* on yield spreads in sovereign bond market can be fairly challenging. In times of market distress, investors demand for safer and liquid assets rises. Hence, flight-to-quality and flight-to-liquidity could occur simultaneously and be positively correlated causing the differentiation between those two effects to be quite difficult (see, Beber et al. (2009)). Barrios et al. (2009) pointed to an additional difficulty, showing that quality and liquidity may also be negatively correlated. For example, the growth in the supply of government bonds decreases liquidity premium and/but increases credit premium (due to a higher default risk), at once.

The results of previous empirical studies regarding the effects of liquidity risk are rather heterogeneous. Due to the many-sided issues of the liquidity concept, various different measures of liquidity risk were used in previous contributions.^3^ Most widely used liquidity proxies are the bid-ask spread and the outstanding amount of public debt. Prior empirical work can be found in Amihud and Mendelson (1986). They model the effects of liquidity on asset returns showing that the bid-ask spread is a good proxy for capturing the transaction cost. Following up on this work, Amihud and Mendelson (1991) and Fleming (2003) confirm the importance of the bid-ask spread as a liquidity variable in the US Treasury market.

Codogno et al. (2003), Geyer et al. (2004), and Pagano and von Thadden (2004) analyze yield spreads of government bonds in the euro area, mostly focusing on the early years of monetary unification. Geyer et al. (2004) find no significant effect of liquidity variables on yield spreads, whereas Codogno et al. (2003) and Pagano and von Thadden (2004) find that liquidity plays at best only a minor role in explaining the yield differences in EMU government bond markets. Bernoth et al. (2012) and von Hagen et al. (2011) focus on bond market size to proxy liquidity risk since the bid-ask spread does not exist for yield-at-issue and the issue size turned out to be highly insignificant. Their results show that while liquidity historically had an effect on yield spreads, this effect disappears with the start of EMU. They explain this finding with the fact that the euro-denominated government bond market became much larger and more integrated after monetary unification. Bernoth and Erdogan (2012) analyze the determinants of sovereign bond yield spreads for 10 EMU countries covering the period from Q1/1999 to Q1/2010. They apply a semiparametric time-varying coefficient model and find that liquidity never played a significant role in explaining yield spreads. Maltritz (2012) uses a Bayesian model averaging approach and finds only weak evidence of the role of liquidity.

In contrast, Gomez-Puig (2006, 2008), Jankowitsch et al. (2006), Beber et al. (2009), Haugh et al. (2009), Manganelli and Wolswijk (2009), Favero et al. (2010), and Favero and Missale (2012) find that liquidity risk is an important factor in explaining yield spreads in European government bond markets. Gomez-Puig (2006) uses daily data over the period between January 1996 and March 2001 including all EMU countries (except Luxemburg and Greece) to analyze the start of the EMU and its effects on the yield spreads in European government bond markets. She uses both bid-ask spreads and the overall outstanding amount of public debt securities to measure liquidity risk. She shows that liquidity is an important determinant of yield spreads in the pre-EMU as well as in the EMU period and that the relative market size of public debt matters. She underpins these results in a subsequent study (Gómez-Puig (2008)). Jankowitsch et al. (2006) employ two different procedures using daily data for the period from January 1999 to March 2001. They show that liquidity effects are more pronounced in smaller countries and are not able to explain the size of yield spreads between issuing counties. These results are confirmed by Favero and Missale (2012). They find that the effect of liquidity is more important for smaller countries, such as Finland and the Netherlands.

Favero et al. (2010) provide a simple model with endogenous liquidity demand, focusing mostly on the interaction between liquidity and aggregate risk. The main result shows that the interaction term has a negative impact on yield spreads, meaning that the liquidity premium tends to be lower when aggregate risk is higher. On the other hand, Beber et al. (2009) find that although the credit premium typically plays the most important role in explaining the valuation of the yield spreads, in times of higher market uncertainty, the investors chase liquidity, not quality. Manganelli and Wolswijk (2009) confirm these results, suggesting that in periods when interest rates are high, the liquidity premium can explain even up to a half of the total yield spreads. The more recent literature focusing extensively on the effects of the financial and sovereign debt crises in Europe (see, e.g., Sgherri and Zoli (2009), De Santis (2012), Gerlach et al. (2010), Giordano et al. (2012), Afonso et al. (2015a)), has in general reached similar findings regarding the role of liquidity. The liquidity premium plays (albeit inferior) a significant and a non-negligible role in explaining yield differentials in European bond markets, which increases intensively in periods of high market turmoil.

There are only a few studies that analyze sovereign bond yield spreads in advanced economies and therefore also in the UK. The previous literature usually studied sovereign risk in emerging markets or has dealt with euro area counties, particularly since the onset of the financial and, later on, the sovereign debt crisis. Moreover, the majority of those analyses are conducted in a panel setup (see, e.g., Ardagna et al. (2007), Gruber and Kamin (2012), and Capelle-Blancard et al. (2019)); thus, very little is known about the determinants of sovereign risk premiums in the UK government bond market. Ilmanen (1995) uses monthly data to examine the predictability of excess government bond returns in six advanced economies (US, Canada, Japan, France, Germany, and UK) for the period from January 1978 to June 1993. He finds that the excess bond returns of the respective countries are highly correlated over time. Looking at the same sample of the countries (without France), Barr and Priestley (2004) confirm this finding, showing that three-quarters of excess bond returns is related to international bond market risk. Studying yield comovements of EMU countries, the UK, the USA, and 16 German Länder, Schulz and Wolff (2008) find government bond market integration already started, not only in euro area countries but also in the UK, in the early to mid-1990s. Using a novel conceptual framework for measuring financial integration, Hoffmann et al. (2020) likewise confirm this finding.

Dungey et al. (2000) perform a factor analysis of long-term bond spreads by decomposing international interest rate spreads into national and global factors. They examine bond yield spreads between five countries (Australia, Canada, Japan, Germany, and the UK) and the USA using weekly data between January 1991 and April 1999. Although the results show that for the UK and Germany, individual country factors exhibit strong effects on bond yield spreads, the common world factor accounts for nearly 90% of total volatility in spreads. Analyzing the effects of the recent financial crisis, Caceres et al. (2010) use daily data from mid-2005 through early 2010, for a total of over 1,000 daily observations. Although focusing on ten euro area countries in their extended sample, they also assess the yield spreads of the USA, Japan, Sweden, and the UK. One important finding in their analysis is that the UK benefits from risk aversion, suggesting that in the times of higher uncertainty, UK government bonds enjoy the reputation of a safe asset. The fundamentals also seem to play a significant role, although the coefficients are rather small. D’Agostino and Ehrman (2014) confirm these findings for the UK, indicating that the coefficients for liquidity and a fiscal variable indeed have the expected sign and are statistically significant; however, the magnitude of the coefficients suggests that these effects are much smaller than for selected euro area countries. In their study, and in contrast to Caceres et al. (2010), they find no beneficial effects of risk aversion on UK government bonds, since a higher risk aversion increases the yield spreads. The difference in the results could be due to the fact that different benchmark yields as a proxy for the risk-free rate are used. While Caceres et al. (2010) use the yield on 10-year swap rates, D’Agostino and Ehrmann (2014) use the German Bund yield. These findings underline the importance of the benchmark used in the analysis.

## Data description

This section presents the data used for estimating risk premiums in sovereign bond markets. The analysis focuses on the UK and nine of the largest euro area economies (Austria, Belgium, France, Germany, Italy, Ireland, Netherlands, Portugal, and Spain). The frequency of data is daily, covering the period from October 1, 2014, to March 29, 2019.^4^

Starting the empirical analysis from October 2014 facilitates avoiding consequential effects of the sovereign debt crisis in Europe. March 29, 2019, is chosen, since the UK was previously supposed to leave the EU by the end of that day, before additional extensions were arranged. Assuming that the market pricing behavior has changed with the UK’s decision to leave the EU, the sample is additionally sub-divided into the period before the Brexit referendum and the period after the Brexit referendum. The dependent variable in this empirical approach is the daily 10-year sovereign bond yield spread relative to the respective 10-year OIS rate. Before performing a rigorous econometric analysis explaining the yield spreads, it is worth presenting graphically the government bond spreads and determining variables for the particular period.

**Fig. 1:**
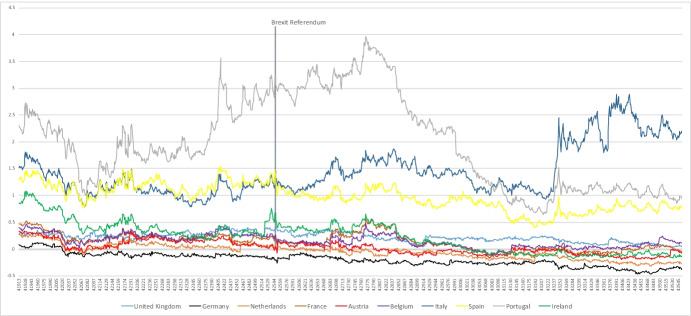
Sovereign bond yield spreads (daily, Oct. 2014 to March 2019). Source: Datastream, own calculation

Figure 1 presents the 10-year government bond yield spreads for the selected countries. From the figure, it is immediately apparent that the Brexit referendum result indeed had an impact on risk premiums of the majority of countries considered. It seems that there are no or only modest visible effects on the risk premiums of Germany, the Netherlands, and Austria. It is also noteworthy that Italy is somewhat of a special case, since it has a considerably higher risk premium at the end of the sample relative to the other countries. Data for the 10-year government benchmark bond yields and the respective 10-year OIS (OIS stands for overnight index swap) rates are provided by Datastream.

**Fig. 2:**
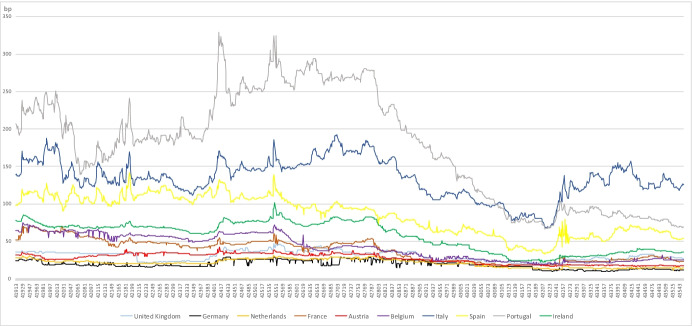
Credit default swap premium on 10-year government bond (daily, Oct. 2014 to March 2019). Source: Datastream, own representation

**Fig. 3:**
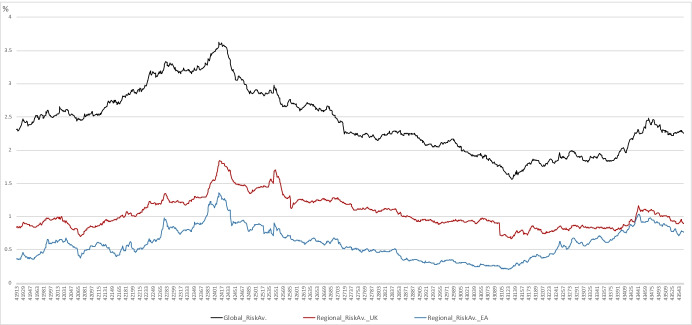
Risk aversion variables by comparison (daily, Oct. 2014 to March 2019). Note: Global risk aversion is calculated as Moody’s US Baa corporate bond yield relative to the yield on 10-Year Treasury; regional risk aversion is calculated as a spread between BBB and AAA corporate bond index using iBoxx £ corporate for the UK and iBoxx € corporate for the EA countries, respectively. Source: Datastream, Federal Reserve Bank of St. Louis, own calculation and representation

In order to approximate the credit default risk of the selected country, the corresponding 10-year credit default swap (CDS) premium on government bonds is used. Bond market size discounted by the yield of the corresponding bond is used as a proxy for the liquidity risk (see Schuknecht et al. (2009)). To consider investors’ risk aversion, a newly developed regional variable is used. Regional risk aversion is calculated as the spread between BBB and AAA corporate bond index, by using iBoxx corporate indices as calculated and provided by IHS Markit. iBoxx £ corporate and iBoxx € corporate indices are used when calculating the regional risk aversion for the UK and euro area countries, respectively. All above data are extracted from Datastream. As a proxy for a more generally used international risk aversion, the US corporate credit spread represented by Moody’s US Baa corporate bond yield relative to the yield on 10-Year Treasury bonds is used. Corresponding data are obtained from the Federal Reserve Bank of St. Louis. Figures 2 and 3 present the time series performance of CDS premiums and respective risk aversion variables (see Appendix).

## Empirical results

This section introduces a simple estimation model to analyze sovereign bond yield spreads in the UK and selected euro area countries and whether the Brexit referendum result affected the respective risk premiums and whether there are any changes in risk pricing after the announcement of the referendum result.

### Methodology

The choice of yield spread determinants is mainly based on the theoretical background and on the existing literature in this field. Estimation is conducted via OLS using daily data, over the period from 1 October, 2014, to 29 March, 2019.^5^ The following simple regression model has been run for each country separately, as the key focus is not on an average effect, but rather on the effect in each country individually:

1 $$\Delta Spr_{j,t} = \alpha + \gamma brexit_{t} + \beta_{1} \Delta liquidity_{j,t} + \beta_{2} \Delta cds_{j,t} + \beta_{3} \Delta regional\_riskav._{j,t} + \Delta Spr_{j,t - 1} + \varepsilon_{t}$$

where $\Delta Spr_{j,t}$ is the dependent variable of interest and represents the daily change of the risk premium in country *j*. The risk premium in country *j* is defined as the difference between a 10-year government benchmark bond yield and a respective 10-year OIS rate. When studying a sovereign bond market, a first crucial issue is the definition of the yield spread and a second is the identification of the determining variables.^6^ The yield spread should be founded on the pricing of risk of an examined asset relative to the risk-free rate. Typically, the yield on a German bond is used as a benchmark for the risk-free rate.^7^ Following the argumentation in ECB (2014b), this analysis departs from earlier studies by using the OIS rate as a risk-free rate but is by no means the first not to use the German bond as a benchmark.^8^ Ejsing et al. (2015) quantify liquidity and credit premiums in German and French government bond yields by using the OIS rate as a risk-free rate. They argue that the OIS rate is the best directly observable measure of the risk-free rate, especially in periods of higher financial market uncertainty.

Given the questions raised by this analysis, using the OIS rate as a benchmark offers some advantages. Firstly, it allows analyzing the risk premium in the German government bond market itself. Since the German Bund is generally used as a benchmark, not much is known about the associated risk premium and its determinants. Secondly, analyzing the effects of the Brexit referendum result on the risk premium in the UK is one of the key aspects of this study. Using the OIS rate and not the German Bund as a benchmark has the advantage of avoiding exchange rate aspects between the British pound and euro that arises through the comparison of bonds denominated in different currencies (see, e.g., Favero et al. (1997) and D’Agostino and Ehrmann (2014)). Thirdly, higher demand for German bonds as a safe haven asset could remarkably influence the risk premiums of selected countries. De Santis (2012) shows that the yield spreads of Austria, Finland, and Netherlands were mainly driven by a higher demand for German sovereign bonds during the recent financial crisis. Studying the yield spread of German relative to US bonds, D’Agostino and Ehrmann (2014) find that in times of increasing international risk aversion, Germany’s safe haven status even improves relative to that of the USA. Using the OIS as a benchmark rate enables this study to avoid the effects of Germany’s safe haven status on the risk premiums in selected countries.

Brexit is an independent variable of interest. This vector is an event dummy variable that is associated with the announcement of the referendum result. This dummy variable takes the value of one on June 24, 2016, and zero elsewhere. In the majority of the previous studies, Brexit is related to lower expected future GDP growth rates due to lower aggregate productivity (see Welfens and Hanrahan (2018) and Belke et al. (2018)). However, the magnitude of the estimated effects depends largely on the outcome of the upcoming negotiations between the UK and the EU regarding their future trade relationship. While the major effects are expected to unfold in a medium- and long-term perspective, the UK’s decision to leave the EU has already generated negative effects on the UK economy (see Bank of England (2018, 2019) and Bloom et al. (2019)). However, it is not only the economy of the UK which is expected to be affected, large implications are also expected for the economies of the remaining EU member states (see IMF (2016, 2018), DGIP (2017), Welfens (2017), and Mion and Ponattu (2019)).^9^ Given the expected effects on the real economy, substantial uncertainty about the upcoming talks on the future relationship between the UK and the EU, and the concerns about future fiscal stance, the Brexit variable is associated with a positive effect on the yield spreads and therefore with increasing risk premiums.

The variable $liquidity_{j,t}$ captures the impact of liquidity risk on the risk premiums. In general, investors holding less liquid assets would require a higher return as a financial compensation for bearing the additional risk. This implies higher yields and therefore higher yield spreads for less liquid bonds. Following the literature (e.g., Gomez-Puig (2006), Schuknecht et al. (2009), and D’Agostino and Ehrmann (2014)), the outstanding amount of debt securities is used to control for liquidity risk.^10^ A larger market size for a given security implicates positive effects on the liquidity, due to the lower information cost, higher trade frequency, and a relative higher number of investors. Therefore, a negative impact of the liquidity variable on the risk premium is expected.

In order to account for credit risk, the corresponding 10-year credit default swap premium on government bond ($cds_{j,t}$) is used.^11^ A CDS is a derivative contract that provides the buyer of the contract with a protection against a negative “credit event” of the issuer of the underlying asset (see Longstaff et al. (2011), Aizenman et al. (2013)). A CDS premium should incorporate all available information on the present as well as on the future expected fiscal stance of each country (Klose and Weigert (2014)). Thus, sovereign CDS represents an excellent market-based direct measure of credit risk in the risk premium. An increasing CDS spread is associated with rising credit risk. Hence, a positive impact of the CDS variable on the risk premium is expected.

In the previous literature, a common factor is considered a major driving factor of the yield spreads in government bond markets, which again is strongly associated with international risk aversion. Therefore, risk aversion plays a special role in explaining yield differentials. Due to the complex interconnection of the financial markets worldwide, globalization, and the size of the US financial market, its role as an international risk indicator is understandable. Nevertheless, the question arises as to whether there might be indicators that are more appropriate (see Manganelli and Wolswijk (2009)). Bernoth et al. (2012) already put forward the idea of a corporate bond spread for the complete euro area. However, due to the lack of data, they could not pursue this idea any further.

This work follows on from the previous consideration by developing a pure corporate bond spread for the EA and the UK, respectively. The novel regional risk aversion ($regional\_riskav._{j,t}$) represents the difference between BBB and AAA corporate bond yields in the considered markets. AAA-rated bonds are used as a representative of the highest credit quality and BBB-rated bonds as a representative of a lower credit quality, since this is the lowest investment grade category. Following Dötz and Fischer (2010), the corporate bond spread is associated with the financing conditions for firms and the macroeconomic growth outlook, which in the end would have an effect on a country’s risk premium. Furthermore, risk aversion may have an impact on the risk premium because of the reputation of the issuing government or because of higher uncertainty of future economic policy (see Codogno et al. (2003)). To sum up, a higher risk aversion implicates a higher demand for safe assets and rising yield spreads. Thus, a positive impact of the regional risk aversion on risk premiums in the UK and the EA countries is expected.

As shown by Attinasi et al. (2010) and Gerlach et al. (2010), sovereign bond spreads can be highly persistent. In order to provide robust estimates of the effects of independent variables and to eliminate possible remaining autocorrelation in the residuals, a lagged dependent variable is included in the regression model (see Wilkins (2018)).^12^ The determining variables are not entered in relative terms, since no country is used as a benchmark. This has several further advantages (see D’Agostino and Ehrmann (2014)). The risk premium, CDS spread, and regional risk aversion are expressed as percentage point changes. The outstanding amount of debt securities is expressed in percentage rates of change. Table 1 summarizes the description of the explanatory variables and gives the expected sign of each.

**Table 1:** Variable description and expected sign

Variable	Description	Expected sign
$\Delta Spr_{j,t}$	*Change in the spread between the yield on 10-year government bond and respective 10-year OIS rate*	
$brexit_{t}$	*Dummy variable for the United Kingdom European Union membership referendum*	+
$\Delta liquidity_{j,t}$	*Change in the outstanding amount of debt securities of the issuer country*	−
$\Delta cds_{j,t}$	*Change in credit default swap premium on government bonds*	+
$\Delta regional\_riskav._{j,t}$	*Change in BBB relative to AAA corporate bond index in respective country*	+
$\Delta global\_riskav._{j,t}$	*Change in Moody’s seasoned Baa corporate bond yield relative to yield on 10-Year Treasury*	+

Own representation

### Direct effects of Brexit referendum announcement

The next section investigates whether the announcement of the Brexit referendum result had an impact on risk premiums in the UK and the EA countries, respectively. The benchmark specification also includes the liquidity premium, the CDS premium, the regional risk aversion measurement, and the lagged dependent variable. The estimation results are reported in Table 2.

**Table 2:** The impact of the Brexit referendum on risk premium

Variable	UK	NLD	GER	FRA	AUT	BEL	ITA	ESP	PRT	IRL
Constant	− 0.0001	− 0.0004	− 0.0004	− 0.0005	− 0.0003	− 0.0003	0.0005	− 0.0004	− 0.0009	− 0.0006
	(0.0004)	(0.0004)	(0.0004)	(0.0006)	(0.0005)	(0.0005)	(0.0012)	(0.0009)	(0.0012)	(0.0006)
Brexit	0.0540***	0.0241	− 0.0017	0.0422***	0.0184**	0.0343***	− 0.0449**	0.0708**	0.1024***	0.0493**
	(0.0129)	(0.0215)	(0.0055)	(0.0071)	(0.0077)	(0.0075)	(0.0202)	(0.0283)	(0.0251)	(0.0228)
Liquidity	− 0.0933***	− 0.1396 ***	− 0.0292*	− 0.0736***	− 0.2497***	− 0.1282***	− 0.1625***	− 0.4052***	− 0.6420***	− 0.4004***
	(0.0024)	(0.0034)	(0.0162)	(0.0209)	(0.0515)	(0.0329)	(0.0429)	(0.1397)	(0.1688)	(0.0580)
CDS	0.0005	0.0016*	− 0.0002	0.0007	− 0.0006	0.0005	0.0103***	0.0039***	0.0055***	0.0037**
	(0.0005)	(0.0009)	(0.0003)	(0.0006)	(0.0009)	(0.0004)	(0.0012)	(0.0012)	(0.0011)	(0.0017)
Regional risk av	0.0390	0.0316	− 0.2053***	0.0841*	0.1182*	0.1408***	0.3206	0.5546***	0.7553***	0.4545***
	(0.0240)	(0.0245)	(0.0393)	(0.0475)	(0.0639)	(0.0475)	(0.2240)	(0.1594)	(0.1672)	(0.0812)
Spread(t-1)			− 0.2533***	− 0.1857***	− 0.2350***	− 0.1938***	− 0.1187**	− 0.1225***	− 0.0462**	− 0.1158***
			(0.0315)	(0.0297)	(0.0330)	(0.0346)	(0.0543)	(0.0334)	(0.0225)	(0.0276)
AR(1)	− 0.3515***	− 0.3056***								
	(0.0205)	(0.0175)								
No. Obs	1172	1172	1171	1171	1171	1171	1171	1171	1171	1171
*R*-squared	0.28	0.26	0.15	0.12	0.22	0.21	0.41	0.37	0.53	0.38
Adjusted *R*-squared	0.27	0.26	0.15	0.11	0.21	0,20	0.41	0.36	0.53	0.37

Standard errors in parentheses; ***, **, and * denote significance at the 1%, 5%, and 10% level, respectively

The first lag of the dependent variable is highly statistically significant for all countries indicating a strong persistence in the risk premium data in sovereign bond markets. The proxy for market liquidity is likewise highly significant for all countries. The results show that this variable has, as expected, a negative impact on yield spreads, which is consistent with the literature (see Beber et al. (2009), Gomez-Puig (2006), and Schuknecht et al. (2009)). Hence, an increase in market liquidity is associated with a declining risk premium.

The CDS spread variable is statistically significant in five out of ten countries with the predicted positive sign. The coefficient is largest for Italy, whereas, for example, for Germany and Austria, no significant effects are found. The results indicate that the effect of CDS premiums on yield spreads is even stronger for the countries with a higher risk of default, confirming the previous findings of Klose and Weigert (2014) and Barrios et al. (2009). Moreover, the regional risk aversion variable is statistically significant in seven countries. With the exception of Germany, the sign is positive, indicating that a worsening of the economic climate increases the risk premium of the respective country. In Germany, by way of contrast, rising risk aversion leads to a lower risk premium. These results confirm the safe haven status of the German Bund in international financial markets (see, e.g., Bernoth et al. (2012), De Santis (2012), and Arghyrou and Kontonikas (2012). For the UK, the Netherlands, and Italy, no statistically significant effects are found.

The Brexit-event dummy variable has a statistically significant positive impact on risk premiums in almost all analyzed countries. These results confirm the expectation that the announcement of the Brexit referendum result is associated with increasing risk premiums in the UK and selected EA countries because of future expected effects on those economies.^13^ Surprisingly, the empirical results suggest a negative impact of the Brexit variable on the risk premium in Italy. Since the direct short-term effect of the referendum announcement is measured, this finding could be driven by a clear lead of the CDS prices over bond yield spreads in the price discovery process (see Blanco et al. (2005), Dötz (2007), Dötz and Fischer (2010), and Fontana and Scheicher (2010)). For Germany and the Netherlands, no statistically significant effects are found, indicating that Dutch, like German, sovereign bonds are perceived to be relatively safe assets (see de Jong (2018)).

### Time-varying aspects

The importance of credit quality, liquidity, and risk aversion in explaining the risk premium is likely to be affected by the state of economy, the macroeconomic growth outlook, and responses to changes in the level of uncertainty (D’Agostino and Ehrmann (2014)). Hence, the influence of the determining variables differs not only by country, but it can also vary over time. Several empirical papers already find and confirm regime-switching dynamics in yield spreads’ determination in the European government bond market due to the recent financial and sovereign crisis (see, for example, Barrios et al. (2009), Gerlach et al. (2010), Favero and Missale (2012), and De Grauwe and Ji (2013)).

In this work so far, the possible time variation in investors’ risk sensitivity and the pricing of risk due to the Brexit referendum result is not taken into account. Bloom et al. (2018, 2019) find that the UK’s decision to leave the EU in the June 2016 referendum has generated a large, broad, and long-lasting increase in uncertainty. Therefore, since it can be assumed that Brexit has a lasting impact on the coefficients of the estimate, Eq. (1) is estimated separately for the period prior to and the period after the announcement of the referendum result. The two estimation periods thus extend from October 1, 2014, to June 23, 2016, and from June 24, 2016, to March 29, 2019, respectively, to consider the potential changing evaluation of the determinants of risk premiums. The results are reported in Table 3.

**Table 3:** Time-varying effects of the Brexit referendum

Pre-referendum period	(October 1, 2013, to June 23, 2016)									
Variable	UK	NL	GER	FRA	AUT	BEL	ITA	ESP	PRT	IRL
Constant	0.0002	− 0.0005	− 0.0005	− 0.0008	− 0.0001	− 0.0007	− 0.0017	− 0.0003	− 0.0015	− 0.0012
	(0.0008)	(0.0008)	(0.0007)	(0.0009)	(0.0008)	(0.0009)	(0.0017)	(0.0014)	(0.0021)	(0.0012)
Liquidity	− 0.0767***	− 0.1563***	− 0.0222	− 0.0624	− 0.3931***	− 0.1175*	− 0.1065***	− 1.2366***	− 0.8973*	− 0.4058***
	(0.0049)	(0.0074)	(0.0334)	(0.0467)	(0.0528)	(0.0607)	(0.0313)	(0.1821)	(0.4794)	(0.0621)
CDS	0.0006	0.0023	− 0.0006	− 0.0008	− 0.0014	0.0004	0.0080***	0.0042***	0.0063***	0.0098***
	(0.0012)	(0.0023)	(0.0005)	(0.0005)	(0.0012)	(0.0006)	(0.0011)	(0.0015)	(0.0016)	(0.0026)
Regional risk av	− 0.0317	0.0887**	− 0.1792***	0.1027*	0.2262***	0.1504**	0.3956*	0.6229***	0.6054***	0.4779***
	(0.0506)	(0.0423)	(0.0512)	(0.0599)	(0.0528)	(0.0722)	(0.2180)	(0.1873)	(0.1675)	(0.0940)
Spread(t-1)			− 0.2651***	− 0.1879***	− 0.1579***	− 0.2035***	− 0.2639***	− 0.1524***	− 0.0521*	− 0.1297***
			(0.0522)	(0.0510)	(0.0526)	(0.0568)	(0.0534)	(0.0361)	(0.0272)	(0.0363)
AR(1)	− 0.3643***	− 0.3040***								
	(0.0356)	(0.0318)								
No. Obs	451	451	450	450	450	450	450	450	450	450
*R*-squared	0.27	0.22	0.14	0.08	0.23	0.17	0.42	0.62	0.66	0.41
Adjusted *R*-squared	0.27	0.21	0.13	0.07	0.22	0.16	0.41	0.62	0.65	0,40
Post-referendum period	(June 24, 2016, to March 31, 2019)									
Variable	UK	NL	GER	FRA	AUT	BEL	ITA	ESP	PRT	IRL
Constant	− 0.0001	− 0.0003	− 0.0003	− 0.0002	− 0.0000	− 0.0001	0.0018	− 0.0004	− 0.0015	− 0.0002
	(0.0004)	(0.0004)	(0.0005)	(0.0007)	(0.0006)	(0.0006)	(0.0015)	(0.001)	(0.0014)	(0.0006)
Liquidity	− 0.1290***	− 0.1311***	− 0.0307*	− 0.0796***	− 0.2186***	− 0.1374***	− 0.2361***	− 0.2569**	− 0.5052***	− 0.3910***
	(0.0071)	(0.0035)	(0.0184)	(0.0216)	(0.0528)	(0.0319)	(0.0905)	(0.1040)	(0.1280)	(0.1149)
CDS	0.0009**	0.0018**	− 0.0001	0.0019**	0.0007	0.0008**	0.0130***	0.0025***	0.0031**	0.0027*
	(0.0004)	(0.0008)	(0.0003)	(0.0009)	(0.0008)	(0.0004)	(0.0020)	(0.0009)	(0.0014)	(0.0014)
Regional risk av	0.1227***	− 0.0192	− 0.2597***	0.1022	0.0578	0.1532**	0.1670	0.7181***	1.0572***	0.4081***
	(0.0203)	(0.0274)	(0.0550)	(0.0865)	(0.1111)	(0.0753)	(0.4468)	(0.1632)	(0.2449)	(0.1423)
Spread(t-1)			− 0.2452***	− 0.1903***	− 0.2786***	− 0.1928***	− 0.0419	− 0.1033**	− 0.0460	− 0.1257***
			(0.0378)	(0.0370)	(0.0419)	(0.041)	(0.0569)	(0.0467)	(0.0342)	(0.0313)
AR(1)	− 0.3408***	− 0.3114***								
	(0.0254)	(0.0258)								
No. Obs	721	721	721	721	721	721	721	721	721	721
*R*-squared	0,30	0.31	0.17	0.15	0.23	0.24	0.47	0.31	0.39	0.35
Adjusted *R*-squared	0,30	0.31	0.16	0.14	0.23	0.23	0.46	0.31	0.39	0.34

Standard errors in parentheses; ***, **, and * denote significance at the 1%, 5%, and 10% level, respectively

The estimation results confirm the time-varying behavior of the risk premium determinants indicating a change in risk pricing after the announcement of the Brexit referendum result. The proxy variable for liquidity became highly statistically significant for all countries in the post-referendum period. This finding confirms that in episodes of increased financial market turmoil, investors are more averse to illiquidity shocks and they respond by switching to more liquid assets (see Beber et al. (2009)). Beyond that, CDS premiums became statistically significant in four countries additionally (the UK, the Netherlands, France, and Belgium). This result shows that, particularly in periods of stressed macroeconomic and financial conditions, the credit risk factor plays a much more important role by determining changes in risk premiums. Considering the regional risk aversion variable, a somewhat more differentiated picture emerges. In the post-referendum period, risk aversion became insignificant for the Netherlands, France, Austria, and Italy. These results indicate that the government bonds of those countries became more reliable assets. The estimated coefficient for Germany remained highly statistically significant with the negative sign and even increased in its magnitude. This result underpins the safe haven status of the German Bund in financial markets, a status that became even stronger after the Brexit referendum. Since the Brexit referendum, the UK is the only country for which the risk aversion variable became statistically significant with a positive sign.

Indeed, the most pronounced change in the behavior of the risk premium determinants can be observed for the UK. Results of estimating the risk premium for the time before and after the referendum show that the proxy variable for credit risk as well as for risk aversion turned significant. In the post-referendum period, both variables became statistically significant with a positive impact on the risk premium in the UK. These results are similar to the results of Belke et al. (2018). Analyzing the effects of Brexit on CDS spreads of nineteen countries, they find that the strongest increase of CDS spreads is observable for the UK. These findings indicate that the Brexit referendum result has an effect on the creditworthiness of the UK.^14^ This might have considerable implications for policymakers in the UK when it comes to taking required actions in order to soften the impact in the aftermath of Brexit. A more aggressive fiscal approach could have further positive effects on credit risk leading to a higher risk premium. In addition, there are highly statistically significant positive effects of the regional risk aversion variable on the risk premium in the UK after the Brexit referendum. This result suggests that there is a substantial change in investors’ risk perception and pricing, indicating that the UK government bond might have lost its reputation as a safe asset that it had enjoyed previously, at least during the financial crisis (see Caceres et al. (2010)). After the Brexit referendum, a higher risk aversion leads to increasing risk premiums. This shift in risk pricing could lead to a higher price of risk (e.g., credit risk), even if the amount of risk has not changed. Lowering London’s status as an international financial center might diminish British sterling’s role as an international currency (see Eichengreen (2019)). Thus, a weakening safe haven status could have extensive consequences for the UK’s financial market and the economy.^15^

## Robustness check

The focus of this section is to examine the robustness of the findings regarding risk aversion as previously reported. As a reminder, this work uses a newly developed regional risk aversion to capture and measure investors’ risk sensitivity and changes in the pricing of risk. The variable is calculated as a yield difference between BBB and AAA corporate bond index in the respective market. Alternative measures of risk aversion are also used in some other studies, for example in Geyer et al. (2004), Haugh et al. (2009), Beber et al. (2009), Gerlach et al. (2010), and Dötz and Fischer (2010). The new regional risk aversion variable used in this study differs from previous ones primarily because it is calculated as a pure corporate yield spread. This has several advantages. Firstly, it enables to avoid any effects from the underlying sovereign bond—for example, safe haven status when using US Treasury bonds or German Bund. Secondly, one important issue is that it can be easily extended and applied to any other country or currency area (e.g., Switzerland).

The most commonly used variable to account for international risk aversion is a spread between US corporate and government bonds. As shown in Fig. 3 (see Appendix), there is strong comovement between the international and regional risk aversion variables. Nevertheless, an unexpected event (i.e., shock) can have different effects on global and regional financial markets, respectively. In order to analyze this, a benchmark model is estimated for the entire sample period once with international and once with the respective regional variables. Moody’s US Baa corporate bond yield relative to yield on 10-Year Treasury bonds is used as a proxy variable for international risk aversion. The results are reported in Table 4.

**Table 4:** Regional versus international risk aversion

Regional risk aversion										
Variable	UK	NL	GER	FRA	AUT	BEL	ITA	ESP	PRT	IRL
Constant	− 0.0001	− 0.0004	− 0.0004	− 0.0005	− 0.0003	− 0.0003	0.0005	− 0.0004	− 0.0009	− 0.0005
	(0.0004)	(0.0004)	(0.0004)	(0.0006)	(0.0005)	(0.0005)	(0.0012)	(0.0009)	(0.0012)	(0.0006)
Liquidity	− 0.0928***	− 0.1394***	− 0.0292*	− 0.0736***	− 0.2495***	− 0.1284***	− 0.1627***	− 0.4061***	− 0.6434***	− 0.4022***
	(0.0024)	(0.0034)	(0.0162)	(0.0209)	(0.0516)	(0.0330)	(0.0430)	(0.1403)	(0.1693)	(0.0582)
CDS	0.0006	0.0018**	− 0.0003	0.0007	− 0.0005	0.0006	0.0103***	0.0040***	0.0055***	0.0043***
	(0.0004)	(0.0009)	(0.0003)	(0.0006)	(0.0009)	(0.0004)	(0.0012)	(0.0012)	(0.0011)	(0.0014)
Regional risk av	0.0633***	0.0408*	− 0.2060***	0.1039**	0.1261**	0.1565***	0.3042	0.5826***	0.8016***	0.4686***
	(0.0225)	(0.0236)	(0.0373)	(0.0494)	(0.0616)	(0.0497)	(0.2198)	(0.1579)	(0.1759)	(0.0841)
Spread(t-1)			− 0.2532***	− 0.1908***	− 0.2370***	− 0.1979***	− 0.1161**	− 0.1273***	− 0.0508**	− 0.1215***
			(0.0315)	(0.0300)	(0.0329)	(0.0344)	(0.0540)	(0.0337)	(0.0225)	(0.0261)
AR(1)	− 0.3463***	− 0.3065***								
	(0.0203)	(0.0175)								
No. Obs	1172	1172	1171	1171	1171	1171	1171	1171	1171	1171
*R*-squared	0.27	0.26	0.15	0.11	0.22	0,20	0.41	0.36	0.53	0.37
Adjusted *R*-squared	0.27	0.26	0.15	0.11	0.21	0,20	0.41	0.36	0.53	0.37
International risk aversion										
Variable	UK	NL	GER	FRA	AUT	BEL	ITA	ESP	PRT	IRL
Constant	0.0000	− 0.0005	− 0.0003	− 0.0006	− 0.0004	− 0.0004	0.0002	− 0.0009	− 0.0012	− 0.0005
	(0.0004)	(0.0004)	(0.0004)	(0.0006)	(0.0005)	(0.0005)	(0.0013)	(0.0009)	(0.0012)	(0.0006)
Liquidity	− 0.0903***	− 0.1438***	− 0.0319*	− 0.0724***	− 0.2386***	− 0.1284***	− 0.1612***	− 0.4562***	− 0.6779***	− 0.4575***
	(0.0024)	(0.0041)	(0.0169)	(0.0219)	(0.0483)	(0.0320)	(0.0408)	(0.1559)	(0.1932)	(0.1012)
CDS	0.0007	0.0020**	− 0.0002	0.0010	− 0.0003	0.0008*	0.0111***	0.0046***	0.0062***	0.0063***
	(0.0005)	(0.0009)	(0.0003)	(0.0007)	(0.0010)	(0.0005)	(0.0009)	(0.0013)	(0.0013)	(0.0016)
International risk av.(t-1)	0.0544**	0.0608***	0.0260	0.0816***	0.0439	0.1136***	− 0.0027	0.0025	− 0.1130	0.0655
	(0.0212)	(0.0207)	(0.0223)	(0.0307)	(0.0340)	(0.0344)	(0.0564)	(0.0552)	(0.0795)	(0.0433)
Spread(t-1)			− 0.2455***	− 0.1842***	− 0.2167***	− 0.1787***	− 0.1149**	− 0.0793**	− 0.0105	− 0.0958***
			(0.0329)	(0.0292)	(0.0346)	(0.0347)	(0.0566)	(0.0372)	(0.0268)	(0.0278)
AR(1)	− 0.3389***	− 0.3002***								
	(0.0221)	(0.0181)								
No. Obs	1069	1069	1069	1069	1069	1069	1069	1069	1069	1069
*R*-squared	0.26	0.26	0.11	0.11	0.21	0.21	0.43	0.34	0,50	0.28
Adjusted *R*-squared	0.26	0.26	0.11	0,10	0,20	0.21	0.43	0.34	0,50	0.27

Standard errors in parentheses; ***, **, and * denote significance at the 1%, 5%, and 10% level, respectively

The first panel of the table shows the results when regional variables are used, while the second panel shows the estimates when the international variable is used. There are several noteworthy findings that should be highlighted. Firstly, the regional risk variable is statistically significant in nine out of ten countries, while the international risk variable is significant only in four countries. From a statistical point of view, this result indicates that using the regional risk variable to capture investors’ risk assessment might be more appropriate, especially when analyzing euro area countries. Secondly, for those countries where additionally the regional risk variable is statistically significant (Germany, Austria, Spain, Portugal, and Ireland), a rise in adjusted *R*-squared is observable. The largest increase in adjusted *R*-squared can be found for Ireland (11 percentage points) and the second largest for Germany (4 percentage points) and then for Portugal, Spain, and Austria. An increase in adjusted R-squared might indicate not only statistical but also economic importance of the regional risk aversion in explaining changes of risk premium in the selected European countries (see Brown (1968)).

## Conclusions

There is an ongoing debate about the potential effects of the UK’s decision to leave the EU. This paper contributes to the literature on the impact of the Brexit referendum on financial markets. It examines whether the announcement of the referendum result has affected the risk premiums in the UK and selected EA countries and whether there are any changes in investors’ risk assessment triggered by the Brexit referendum result. The model includes daily yield spreads data covering the period from October 1, 2014, to March 29, 2019. An important feature of this study is the lead use of the newly developed regional risk aversion variable, which is shown to be an appropriate measure of investors’ willingness to bear a country-specific risk.

The results show that the Brexit referendum had a significant impact on yield spreads leading to higher sovereign risk premiums in the UK and most other selected EA countries. Rising risk premiums may cause government bond yields to increase and force governments to exhibit more fiscal discipline and therefore make it more difficult for policymakers to milden the effects of Brexit and its aftermath. Additionally, the sample is split into the period before and the period after the Brexit referendum, to consider the potential change in the importance of the determinants of risk premiums. The estimation results show that, particularly in periods of stressed macroeconomic and financial conditions, the credit risk factor plays a much more important role by determining changes in risk premiums. This finding implicates that a more aggressive fiscal approach could have further positive effects on credit risk leading again to higher risk premiums. Moreover, the highly statistically significant positive effect of the regional risk aversion variable on the risk premium in the UK after the Brexit referendum indicates that the UK government bond might have lost its reputation as a safe asset. Further weakening of the safe haven status could have extensive consequences for the UK’s financial market and the economy.

Although the underlying study includes nine of the largest EA economies, subsequent research might consider broadening the sample of countries in order to address concerns regarding high heterogeneity amongst the countries of the EA. Furthermore, in analyzing 10-year government bond markets, the study focuses on the effects of Brexit in the longer term. Thus, one might consider incorporating additional maturities, for example 2-year and 5-year maturities, that could help to better understand the dispersion of Brexit effects over time.

In 2019, leader of the Conservative Party Boris Johnson won a resounding electoral victory, giving him a clear mandate with regard to negations on the future of the UK-EU relationship. On January 31, 2020, when the Withdrawal Agreement entered into force, the UK officially withdrew from the European Union. This marked the start of a transition period that ended on December 31 same year, and with the entering into force on January 1, 2021, of the EU-UK Trade and Cooperation Agreement, the UK is now a third country.

Still, this is far from the end of the Brexit process. Despite the broad agreement achieved, there remain clear differences, as the months which followed have shown. Particularly in the area of financial services, no clear agreement could be achieved. Upon leaving the EU, UK financial services lost their passporting rights to service EU clients from the UK and faced the prospect of relying on “equivalence” decisions in order to access the single market. However, the British government no longer assumes that equivalence deals from the EU for financial services will be forthcoming (see, Sunak (2021)).

Therefore, it would be of particular interest for future research to extend the sample in order to include the announcement of the Withdrawal Agreement and the Trade and Cooperation Agreement between the UK and the EU in the analysis. However, one should not overlook the effects of COVID-19 pandemic on international government bond markets. To account for the pandemic effects, incorporating an alternative or additional Brexit variable might be considered.

## Data Availability

All data used in this study are provided by Datastream and/or FRED (Federal Reserve Bank of St. Louis).

## Appendix

Figs. 2 and 3

## Footnotes

1 Recent study by Welfens and Xiong (2019) shows how big intra-EU trade barriers on financial services are. Hence, additional to the traditional factors, barriers to capital flows could also affect intra-EU interest differentials.

2 The basket of variables used to proxy credit risk includes, amongst others, the debt-to-GDP and deficit-to GDP ratios (current and expected), government debt/tax and fiscal deficit/tax ratios (fiscal space), current account balance, ratio of government debt service to current government revenues, real GDP growth, growth rate of industrial production, consumer price inflation, unemployment, real effective exchange rate (REER), terms of trade, trade balance to GDP, openness, credit ratings, and CDS.

3 Liquidity proxies generally used are the bid-ask spread, amount of outstanding public debt, issue size, trading volume, trading frequency, turnover ratio, on-the-run/of the run spread, and some others.

4 The set of countries and the sample period reflect the availability of data.

5 Heteroskedasticity/autocorrelation-robust standard errors are used (OLS-HAC, see Newey and West (1987)).

6 The 3-month OIS rate was added in the regression model, as the determining variable of the short-term interest rate (see Manganelli and Wolswijk (2009)). However, this variable always turned out to be insignificant. Therefore, it was excluded again from the regressions.

7 For a further discussion on benchmark status, please see Dunne et al. (2002, 2007).

8 Several other studies also focus on alternative benchmark interest rates, see, e.g., Klose and Weigert (2014) who use the ECB overnight deposits rate, Pozzi and Wolswijk (2012) use the one week euro currency rate, Caceres et al. (2010) use the yield on a 10-year swap, and Favero et al. (2010) prefer the French OAT as the best choice of a benchmark for the 5-year maturity.

9 The UK is amongst the most important partners of EU member states, and the City of London is, as world No. 1 global financial center, the world leader in fixed-income and derivatives transactions and a major transshipment center for the clearing of euro-denominated trades.

10 Unfortunately, due to the limited access to data, the bid-ask spreads are not included in the estimated regression as an additional proxy for the liquidity.

11 In order to account for possible liquidity bias in the 10-year CDS market, 5-year CDS spreads are also used. Since the CDS coefficients remain roughly the same in sign and magnitude, further estimations are calculated using the 10-year CDS variable.

12 *EViews* allows including ar(1) process in the equation instead of a lagged dependent variable. The decision to include ar(1) process or lagged dependent variable is made on better (higher) Akaike info criterion and Schwarz criterion. This was the case only for the UK and the Netherlands.

13 The statistical significance of the coefficient on a binary dummy depends not only on its “explanatory power” but also on the sample length of the data. Consequently, these results should be interpreted with caution.

14 As a result of the Brexit vote, S&P and Fitch downgraded the sovereign credit rating of the UK to “AA” with a negative outlook on June 27/28, 2016, leaving the possibility of a further downgrade open, depending on the outcome of the Brexit negotiations.

15 For a further discussion on the benefits of safe assets, please see Habib et al. (2020).

## Code availability

Standard software is used (Eviews 9).
